# Developmental diet regulates *Drosophila* lifespan via lipid autotoxins

**DOI:** 10.1038/s41467-017-01740-9

**Published:** 2017-11-09

**Authors:** M. Irina Stefana, Paul C. Driscoll, Fumiaki Obata, Ana Raquel Pengelly, Clare L. Newell, James I. MacRae, Alex P. Gould

**Affiliations:** 1 The Francis Crick Institute, 1 Midland Road, London, NW1 1AT UK; 2 Present Address: JDRF/Wellcome Diabetes and Inflammation Laboratory, Wellcome Centre for Human Genetics, Nuffield Department of Medicine, NIHR Oxford Biomedical Research Centre, University of Oxford, Roosevelt Drive, Oxford OX3 7BN UK; 3 Present Address: Department of Genetics, Graduate School of Pharmaceutical Sciences, The University of Tokyo, 7-3-1 Hongo, Bunkyo-ku, Tokyo 113-0033 Japan

## Abstract

Early-life nourishment exerts long-term influences upon adult physiology and disease risk. These lasting effects of diet are well established but the underlying mechanisms are only partially understood. Here we show that restricting dietary yeast during *Drosophila* development can, depending upon the subsequent adult environment, more than double median lifespan. Developmental diet acts via a long-term influence upon the adult production of toxic molecules, which we term autotoxins, that are shed into the environment and shorten the lifespan of both sexes. Autotoxins are synthesised by oenocytes and some of them correspond to alkene hydrocarbons that also act as pheromones. This study identifies a mechanism by which the developmental dietary history of an animal regulates its own longevity and that of its conspecific neighbours. It also has important implications for the design of lifespan experiments as autotoxins can influence the regulation of longevity by other factors including diet, sex, insulin signalling and population density.

## Abstract (web summary)

The diet consumed during development can have long-lasting effects on adult physiology. Here, the authors show that developmental undernutrition in *Drosophila* extends lifespan by inhibiting the production of toxic lipids, called autotoxins, on the adult body surface.

## Introduction

Epidemiological studies have long suggested that the environment during development influences the risk of adult pathophysiology (reviewed in refs. ^1–3^). There is now substantial evidence from human and rodent studies that early-life nutrition can have a long-term effect (often termed nutritional programming) upon the risks of coronary heart disease, stroke, hypertension, obesity, type 2 diabetes and osteoporosis during adulthood (reviewed in refs. ^4–7^). Similarly, developmental nutrition has been shown to regulate lifespan, increasing or decreasing it depending upon the particular dietary alteration and when it was experienced^8–10^. For example, a maternal low protein diet during suckling increases the longevity of male mice and protects them against the lifespan-shortening effect of a fattening adult diet^9^. Rodent models implicate several mechanisms in the early-life nutritional regulation of adult metabolism, including permanent structural changes to organs, altered cellular ageing and epigenetic regulation of gene expression (reviewed in refs. ^11, 12^). Nevertheless, it is not yet clear the extent to which each of these mechanisms contribute to overall adult physiology. It is also not known whether these or other mechanisms account for the effects of developmental dietary history upon lifespan.

*Drosophila* has proved a useful genetic and physiological model for studying nutrition, growth and metabolism^13–16^. Developmental nutrition is known to influence several aspects of adult metabolism in *Drosophila*. Increasing the sugar:protein ratio in the parental diet elevates glycogen but decreases triglycerides in newly eclosed adult offspring^17^. It is, however, also reported that paternal but not maternal high-sugar diets increase triglycerides in young adult progeny and that this involves epigenetic changes mediated by a H3K9 histone methyltransferase^18, 19^. Lifespan in *Drosophila* and in other species can be extended by dietary restriction (DR) during adulthood (reviewed in refs. ^20–22^). It is also well established that decreased insulin/target of rapamycin (TOR) signalling in the adult can extend lifespan, although the extent to which this contributes to the DR effect remains under investigation (reviewed in refs. ^22–24^). In contrast to studies of adult diet, much less is known about how developmental diet regulates *Drosophila* lifespan. Depriving larvae of dietary yeast, the major protein source, during only the last (third) instar is known to produce adults with a small body size without significantly altering lifespan^25^. It has also been reported that diet or yeast dilution throughout larval development can lead respectively to minor (~7%) or moderate (~25%) increases in lifespan^26, 27^. Hence, there is some evidence that developmental dietary history influences *Drosophila* lifespan but the regimes tested thus far have only generated modest effects and the underlying mechanisms have not yet been identified.

We now establish a new *Drosophila* model for developmental dietary regulation of adult survival and show that it can produce large increases in median lifespan. Combining *Drosophila* genetics and analytical chemistry, we then demonstrate that the underlying mechanism involves long-term suppression of toxic lipids produced by adults, which we name autotoxins.

## Results

### Larval diet regulates adult metabolism and lifespan

**Fig. 1:**
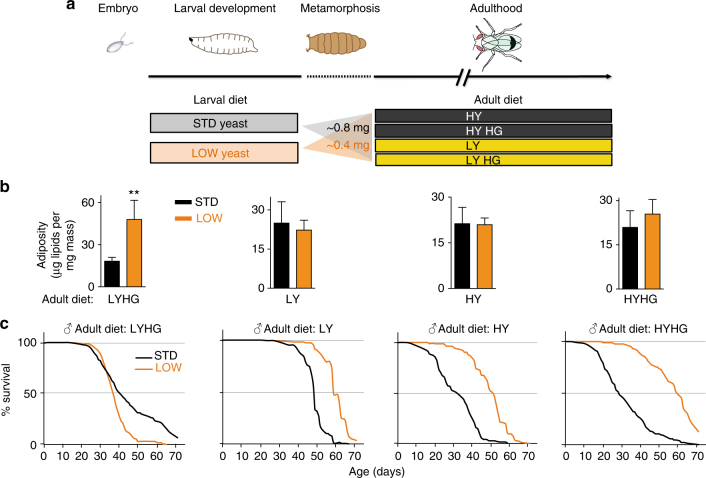
Developmental dietary history alters adult physiology and lifespan. **a** The eight larval-adult dietary combinations used in this study. **b** LOW larval diet increases male adiposity (triglycerides + cholesteryl esters per mg body mass) at 2 weeks post-eclosion on the LYHG adult diet but not on the other three adult diets (in all cases, *n* = 4). **p < 0.01, two-tailed *t*-test. **c** LOW (orange) males survive longer than STD (black) males on three of the four adult diets tested (30 flies per vial). See text for details of adult diets. In this and subsequent figures, error bars show 1 s.d. *n* numbers and the statistical analysis of all survival curves is provided in Supplementary Data 1

The developmental nutritional parameters for the regulation of *Drosophila melanogaster* lifespan were established by rearing a well characterised isogenic strain (*iso31*) on different combinations of larval and adult diets (see Methods section and Fig. 1a). In our standard diet (STD: ~2% yeast, ~6% glucose, ~7% cornmeal), yeast is a major source of amino acids and we found that lowering its concentration throughout larval development by as much as 20-fold (LOW larval diet; ~0.1% yeast) considerably delays development but is nevertheless compatible with adult survival and fertility. The body mass of male and female LOW flies is decreased to ~50% of that of STD controls, with no significant change in the percentage of body water (Fig. 1a, Supplementary Fig. 1a, b, e). STD or LOW animals were transferred after eclosion onto adult diets with yeast at either a low (0.6%; LY) or high (9%; HY) concentration together with glucose at either a standard (6%) or high (40%; HG) concentration. These four adult diets were designed to challenge flies with various suboptimal compositions of macronutrients and the HG diets use an elevated glucose concentration expected to provoke diet-induced stress and metabolic dysfunction in adults^28, 29^. Adult adiposity was similar on seven of the eight larval-adult diet pairs tested but, surprisingly, it increased significantly on the LOW + LYHG combination (Fig. 1b). This indicates that LOW adults have a predisposition to develop high adiposity, but this is only manifested on the LYHG adult diet. This adult diet also appears to compromise survival of LOW flies of both sexes compared to STD controls (Fig. 1c, Supplementary Fig. 1f for females). Importantly, for the other three adult diets, we found that LOW males reproducibly lived longer than STD controls, with median lifespan increases ranging from 20 to 30% on LY, 70 to 90% on HY and up to a striking 145% on the HYHG diet (Fig. 1c, Supplementary Fig. 1c, d). The larval-adult dietary manipulations suggest that low dietary yeast during development is adaptive for longevity on an adult diet that is also low in yeast (LY). Although this resembles a predictive adaptive response^30^ it is not specific as LOW male flies also live longer than STD male flies on the two adult diets that are high in yeast. More generally, we observe that changes in the adult diet tend to alter the median lifespans of both LOW and STD flies but a switch from the most beneficial for longevity (LY) to the challenging HYHG diet strongly decreases the median lifespan of STD but not LOW flies. In summary, depending upon the adult diet, yeast restriction in the larval diet can substantially increase lifespan.

**Fig. 2:**
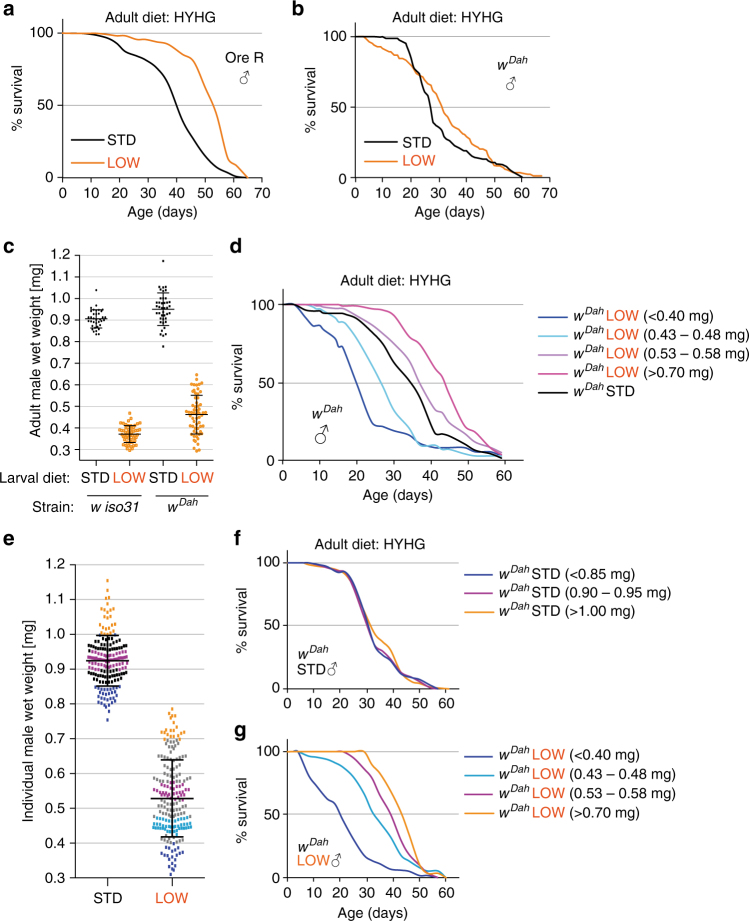
Developmental diet regulates lifespan in heterogeneous genetic backgrounds. **a**, **b** Survival of STD and LOW adult males of *Oregon R* (**a**, OreR) and *white Dahomey* (**b**, *w* ^*Dah*^) strains. **c** Wet body mass of individual STD and LOW adult males at 1 week of age for the *w iso31* and *w* ^*Dah*^ strains. **d** Survival on HYHG of *w* ^*Dah*^ LOW males separated individually into the four indicated weight bins compared to *w* ^*Dah*^ STD males. **e**–**g** Wet body mass at 3 days of age of individual STD and LOW adult males separated into coloured weight bins **e** used for survival curves on the HYHG adult diet **f**, **g**. Note that the weight bin in **e** and its representative survival curve, in **f**, **g** are indicated with the same colour. Error bars show ±1 s.d.

The large developmental-diet induced increase in lifespan of LOW relative to STD flies is not unique to *iso31* males, as it is also observed with *iso31* females and with *Oregon-R* males (Supplementary Fig. 1f, Fig. 2a). However, LOW males of *white Dahomey* (*w* ^*Dah*^), an outbred strain with a high degree of genetic heterogeneity, showed early mortality and barely any overall increase in median lifespan compared to STD (Fig. 2b). *w* ^*Dah*^ males were more variable in body mass than *iso31* and separating individually weighed flies into mass bins revealed that, while STD *w* ^*Dah*^ males in all bins had similar lifespans, LOW *w* ^*Dah*^ males displayed increased median lifespan if their body mass was above ~0.5 mg (Fig. 2c–g). This beneficial effect is reversed in those individuals with more extreme decreases in body mass below ~0.5 mg. Thus, for a subset of the *w* ^*Dah*^ population, as for other strains, a LOW larval diet can substantially increase lifespan. These results demonstrate that the dietary history during development regulates the lifespan of both sexes across a range of different genetic strains and adult diets.

### Larval diet regulates lifespan-shortening adult autotoxins

**Fig. 3:**
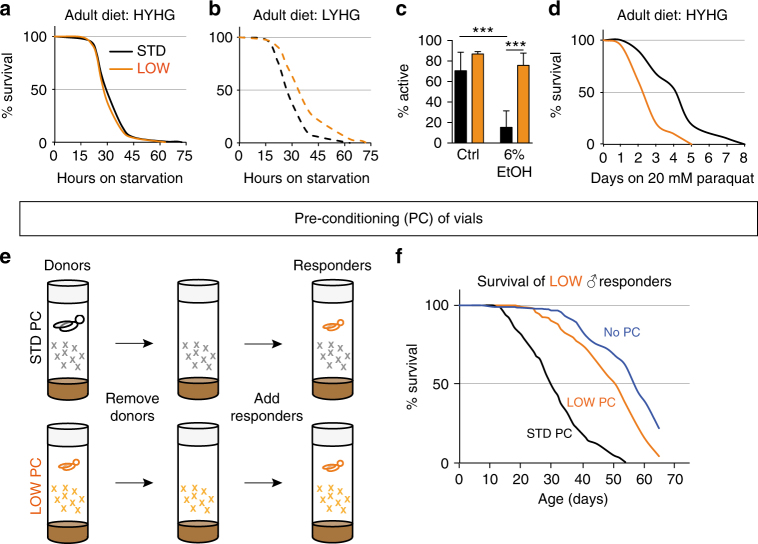
Adult stress resistance and autotoxins are regulated by developmental diet. **a**–**d** Survival of LOW (orange) vs. STD (black) adult males during starvation **a**, **b** 6% ethanol (*n* = 8 vials containing 10–15 males each; climbing ability was scored three times and averaged). **c** or 20 mM paraquat **d**. **e** Vial preconditioning (PC) paradigm for detecting lifespan-shortening autotoxins. **f** The survival of LOW responder males (15 per vial) is shortened in vials preconditioned with STD (black) or LOW (orange) donor males, compared to vials with no preconditioning (*green*). For **c**: ***p < 0.001, ordinary one-way ANOVA followed by Tukey’s multiple comparisons test. Error bars show 1 s.d.

To characterise further the physiology of long-lived LOW flies, we challenged them with several different environmental stressors and toxic xenobiotic compounds. LOW flies have comparable starvation resistance to STD animals when previously fed on HYHG adult diet (Fig. 3a). However, they are more starvation resistant than STD flies when fed on LYHG, the only adult diet that increases the fat stores of LOW relative to STD flies (Fig. 3b). In addition, LOW are more tolerant than STD flies to the effects of 6% ethanol upon locomotor activity (Fig. 3c). In contrast, LOW are more sensitive than STD flies to oxidative stress induced by 20 mM paraquat (Fig. 3d). Together with the earlier lifespan results, this shows that long-term survival outcomes are the result of complex interactions between developmental diet and the subsequent adult environment (diet, stress, toxic compounds).

**Fig. 4:**
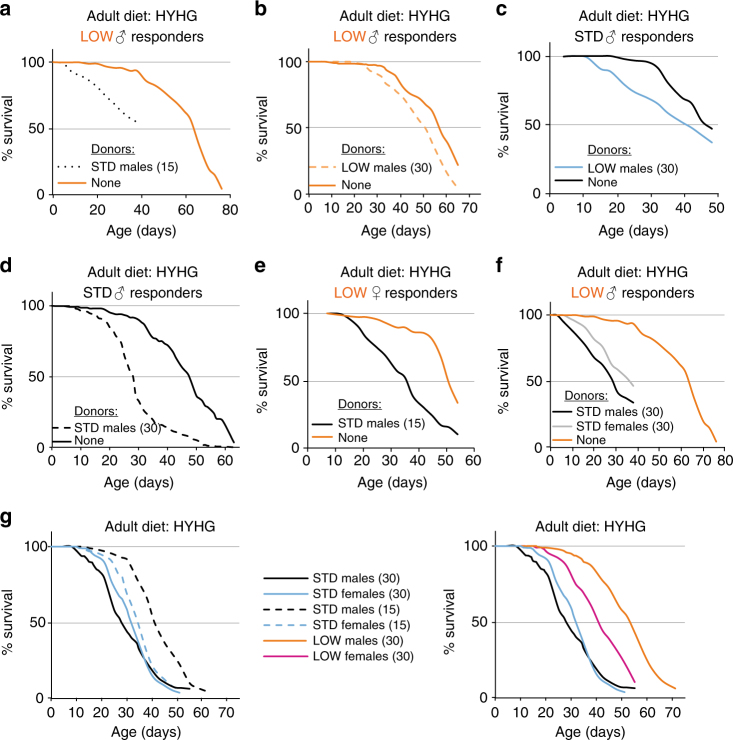
STD and LOW flies of both sexes produce and respond to autotoxins. **a**, **b** Survival of LOW *iso31* responder males is decreased more by vials preconditioned with 15 STD **a** than with an equivalent biomass of 30 LOW **b** males. Survival curves shown in **b** are a subset of those shown in Fig. 3f and are repeated here to facilitate side-by-side comparisons with the data in **a**. **c** Survival of STD responder males (10 per vial) in vials preconditioned with 30 LOW males. **d** Survival of STD responder males (15 per vial) in vials preconditioned with 30 STD males. **e** Survival of LOW responder females (15 per vial) in vials preconditioned with 15 STD males. **f** Survival of LOW responder males (15 per vial) in vials preconditioned with 30 STD males or 30 STD females. **g** The tendency of females to survive longer than males is reversed by housing adults at a lower density (15 per vial; left graph) or by a LOW developmental dietary history (right graph). All graphs use HYHG adult diet and show a survival curve with non-preconditioned control vials (none). To aid comparison, and as the experiments were performed in parallel, the curves with no donors (none) are the same in **a**, **f** and the curves for STD males and females (30 per vial) are also the same in both graphs in **g**

Xenobiotic compounds, such as ethanol and paraquat are not present at toxic levels in the adult diets on which LOW outlive STD flies. Nevertheless, other toxic compounds could be present and so we explored the possibility that flies themselves might condition the environment with endogenously produced substances detrimental to survival (hereafter called autotoxins). A differential production and/or response to these autotoxins could then contribute to lifespan regulation by developmental diet. To test this hypothesis, we developed a vial pre-conditioning paradigm where responder flies are housed in vials that were previously occupied by donor flies (Fig. 3e). This approach revealed that flies do indeed produce autotoxins that shorten lifespan and, importantly, that STD males produce more of them than LOW males (Fig. 3f). This is not simply a “small fly” effect as 15 STD donors shorten LOW responder lifespan considerably more than an equivalent biomass of 30 LOW donors (Fig. 4a, b). This striking finding provides initial evidence that flies condition their environment with lifespan-shortening autotoxins and that the amounts produced depend upon developmental diet such that LOW males produce less, per unit of body mass, than STD males. Vial pre-conditioning experiments were also performed with STD responders, and with donors and responders of the opposite sex (Fig. 4c–f). This showed that both sexes produce autotoxins and also that both sexes are sensitive to each other’s autotoxins. Interestingly, however, females (the larger sex) produce less autotoxin activity per fly than males and therefore generate substantially lower levels of autotoxins per unit of body mass (Fig. 4f). Strikingly, the known tendency for *Drosophila* females to live longer than males^31^ was reversed under conditions that lower autotoxin concentration, such as when LOW flies were used or when the number of STD flies in a vial was decreased (Fig. 4g). Therefore, at least in some conditions, autotoxins are likely to contribute to sex-specific differences in longevity. Together, the vial pre-conditioning experiments suggest that yeast restriction in the developmental diet influences adult flies to produce lower levels of lifespan-shortening autotoxins, which could promote their longevity.

### Autotoxins are alkenes that are developmental-diet dependent

**Fig. 5:**
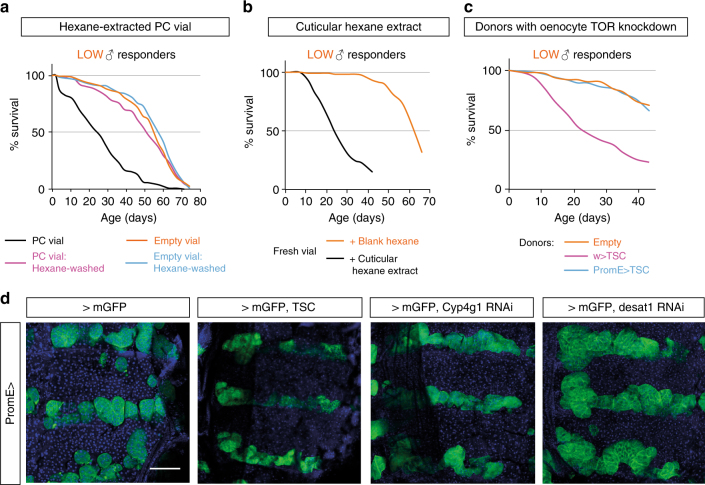
Autotoxins are oenocyte-derived cuticular lipids. **a**, **b** Survival curves for LOW responder males showing that hexane extracts the autotoxin activity from preconditioned (PC) vials **a** or from adult cuticle **b**. **c** Autotoxin activity is not detectable in vials preconditioned with STD male donors overexpressing TSC1 + 2 in oenocytes (*PromE* ^*TS*^ > *TSC*), unlike control (*w* > *TSC*) or non-preconditioned vials (empty). **d** Dorsal oenocytes from 1 week STD males of a control genotype (*PromE* ^*TS*^ > *mGFP*), TSC1 + 2 overexpression (*PromE* ^*TS*^ > ;*mGFP, TSC*), Cyp4g1 RNAi (*PromE* ^*TS*^ > *mGFP, Cyp4g1 RNAi*) or Desat1 RNAi (*PromE* ^*TS*^ > *mGFP, Desat1 RNAi*). Oenocytes and nuclei are marked with mCD8::GFP (green) and DAPI (blue), respectively. Scale bar = 100 μm. In this and subsequent figures, *PromE* ^*TS*^ driven expression was restricted to adulthood (Methods section)

**Fig. 6:**
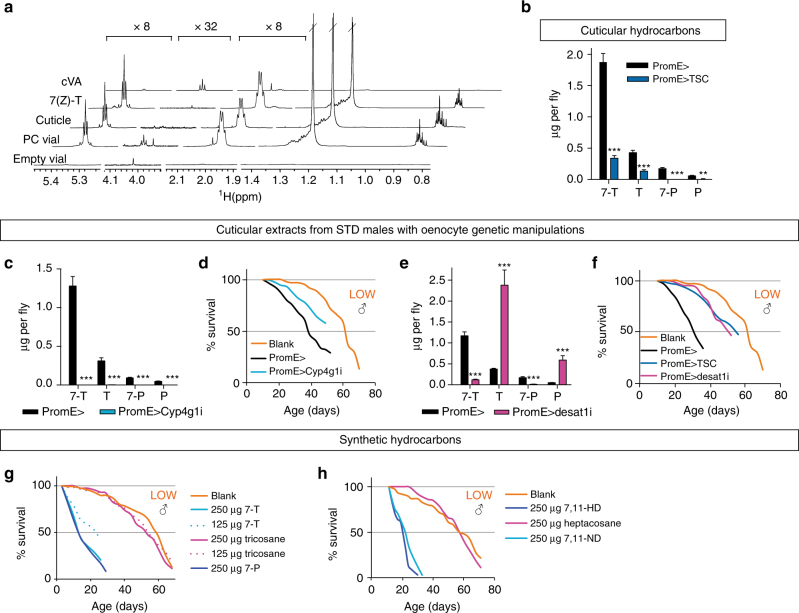
Cuticular alkenes but not alkanes are autotoxic. **a** ^1^H NMR profiles showing that hexane extracts of STD males (Cuticle) and HYHG vials preconditioned with STD males (PC Vial) contain large quantities of 7(Z)-tricosene (7(Z)-T). Profiles from synthetic 7(Z)-T), *cis*-vaccenyl acetate (cVA) and non-preconditioned vials are shown for comparison. **b**–**f** Adult oenocyte genetic manipulations decrease major cuticular hydrocarbons measured by GC–MS (*n* = 6) **b**, **c**, **e** and also decrease the autotoxic activity of cuticular extracts **d**,** f**. Genotypes are as indicated and STD adult males were aged for 3 weeks on HYHG **b**, **e**, **f** or for 1 week on STD adult diet **c**, **d**. Blank curves are the same in **d**, **f**, as these experiments were done in parallel. **g**, **h** Synthetic cuticular alkenes but not alkanes of equivalent chain lengths display autotoxic activity. In **d**, **f**–**h** 15 LOW responder males per HYHG vial were used. For **b**–**e**: **p < 0.01, ***p < 0.001, two-way ANOVA followed by the Bonferroni multiple comparisons test. Error bars show 1 s.d.

To identify the molecules corresponding to autotoxins, we extracted pre-conditioned vials or the body surface (cuticle) of STD flies with different solvents. The majority of autotoxin activity could be extracted from both sources using hexane (Fig. 5a, b). This suggests that autotoxins are lipids that are shed into the vial and are also present on the adult cuticle. As males produce more autotoxins than females, we next investigated the roles of sexually dimorphic adult tissues known to synthesise cuticular or secreted/excreted lipids. We were unable to detect a role for main cells of the male accessory gland in autotoxin production and physiological levels of the ejaculatory bulb pheromone, *cis*-vaccenyl acetate (cVA) had no effect on LOW male lifespan (Supplementary Fig. 2a, b). In contrast, inhibition of the TOR pathway specifically in adult cells called oenocytes (*PromE* ^TS^ > *TSC*, see Methods section) reduced their size and strongly decreased autotoxin activity in vial pre-conditioning assays (Fig. 5c, d). Adult oenocytes form during pupal development and are specialised for the biosynthesis of cuticular hydrocarbons with known roles in pheromone signalling and desiccation resistance (reviewed in refs. ^32–34^). In *Drosophila melanogaster*, both males and females synthesise alkane (saturated) and alkene (unsaturated) hydrocarbons. Alkenes that can function as sex pheromones are monoenes such as 7-tricosene in males, and dienes such as 7,11-heptacosadiene in females (reviewed in refs. ^35, 36^). NMR profiling of hexane extracts from pre-conditioned vials or from STD male cuticles indicated that the majority of the total hexane-soluble signal corresponds to alkenes (Fig. 6a). These data also indicate that *Drosophila* shed a surprisingly large amount of lipids: ~125 μg per preconditioned vial, equating to ~4 μg deposited over 72 h per STD male fly (Methods section). GC–MS analysis of male cuticular extracts showed that both cuticular alkenes and alkanes are strongly decreased by adult oenocyte-specific TOR inhibition (Fig. 6b). Moreover, adult-specific RNAi knockdown of Cyp4g1 (*PromE* ^*TS*^ > *Cyp4g1i*), a P450 oxidative decarbonylase required for the synthesis of cuticular hydrocarbons^37^, depleted alkanes and alkenes, and correspondingly decreased autotoxin activity in STD male cuticular extracts (Fig. 6c, d). Together, the GC–MS measurements and genetic manipulations demonstrate that hydrocarbon synthesis in adult oenocytes is required for the production of high autotoxin activity.

To identify which hydrocarbon species correspond to autotoxins, we knocked down Desaturase 1 (*PromE* ^*TS*^ > *desat1i*), a stearoyl-CoA desaturase required for monoene and diene synthesis^38, 39^. This did not obviously disrupt adult oenocyte morphology but decreased alkenes by ~90%, with a large concomitant increase in alkanes (Figs. 5d, 6e). The lower alkene:alkane ratio is associated with less rather than more autotoxin activity, suggesting that alkenes are more toxic than alkanes (Fig. 6f). To test this directly, LOW adult responders were exposed to physiological quantities of synthetic male-predominant monoenes (7(Z)-tricosene, 9(Z)-tricosene or 7(Z)-pentacosene) or female dienes (7(Z),11(Z)-heptacosadiene or 7(Z),11(Z)-nonacosadiene). Strikingly, fly survival was strongly decreased by vial supplementation with all of the alkenes but not with n-alkanes of equivalent carbon chain lengths, such as tricosane or heptacosane (Fig. 6g, h, Supplementary Fig. 2c). Despite strongly decreasing adult survival, 7(Z)-tricosene did not significantly alter the rate of egg laying by 1–2-week-old females, nor did it delay or kill developing larvae (Supplementary Fig. 2d–f). Thus, 7(Z)-tricosene appears to be a selective rather than an indiscriminate autotoxin. Nevertheless, male and female alkenes of varying carbon chain lengths, including non-volatiles^40^, can all trigger the autotoxic response, indicating that its chemical specificity is broad. Genes encoding contact chemoreceptors of the Pickpocket family, *ppk23* and *ppk29*, are necessary for male-male repulsion and male-female attraction behaviours and, in the case of *ppk23*, reported to mediate the shortening of male lifespan by female dienes^41–43^. Neither of these receptors is, however, required for the lifespan response to autotoxins (Supplementary Fig. 2g, h). Future studies will be needed to pinpoint the physicochemical properties of lipids that confer autotoxin activity and also to identify their mechanism-of-action. However, we note that autotoxin-like survival decreases can also be obtained with hydrocarbons not normally present on the *Drosophila* cuticle, such as two alkanes (heptadecane and squalane) and a branched alkene found in human skin (squalene) but not a very long chain unsaturated fatty acid (nervonic acid, C24:1) (Supplementary Fig. 2i). In summary, genetic analysis and vial supplementations demonstrate that oenocyte-derived alkenes but not alkanes are an important class of biologically relevant autotoxins that shorten lifespan.

**Fig. 7:**
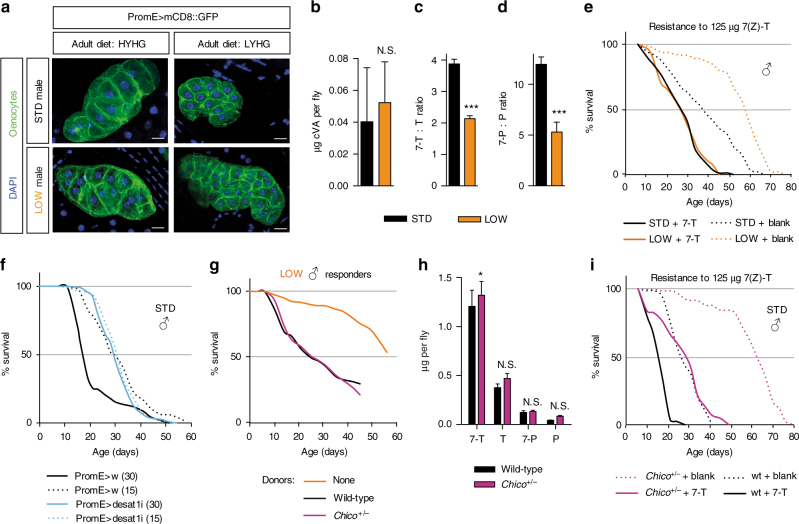
Developmental diet and *chico* regulate autotoxin production and resistance. **a** Ventral oenocytes of 2-week STD or LOW adult males on HYHG or LYHG. Scale bar = 5 μm. **b**–**d** LOW males have a similar amount of *cis*-vaccenyl acetate (**b** cVA) but a lower ratio of 7-unsaturated:saturated C23 (**c** 7-T:T) and C25 (**d** 7-P:P) hydrocarbons than STD males on HYHG at 3 weeks of age (*n *= 6). ***p < 0.001, two-tailed *t*-test. **e** Supplementation with 125 μg 7(Z)-T (7-T) shortens the lifespan of STD and LOW males and abrogates the survival difference observed in unsupplemented (blank) vials. Ten flies were used per HYHG vial. **f** Doubling housing density from 15 to 30 flies per vial decreases the survival of control (*PromE* ^*TS*^ > *w*) but not Desat1 RNAi (*PromE* ^*TS*^ > *Desat1i*) STD males on HYHG. **g**, **h** *chico* ^+/−^ and wild-type STD males produce similar levels of autotoxin activity **g** and cuticular hydrocarbons at 3 weeks of age on HYHG (*n* = 5) **h**. *p < 0.05, two-way ANOVA followed by the Bonferroni multiple comparisons test. **i** Survival of STD *chico* ^*+/*^^−^ and *chico* ^*+/+*^ males (10 per vial) on HYHG supplemented with 125 μg 7(Z)-T (7-T) or with hexane (blank). **b**–**d**, **h**: Error bars show 1 s.d.

**Fig. 8:**
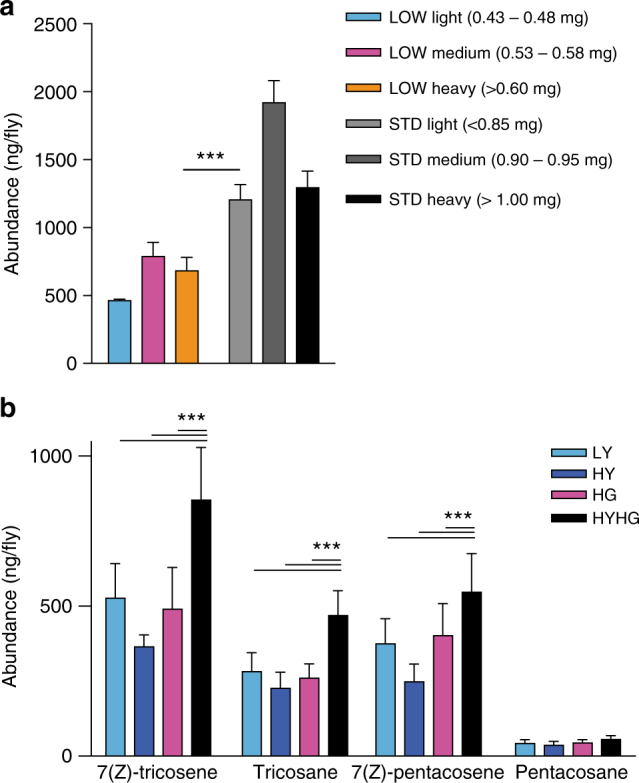
Autotoxin levels are sensitive to both larval and adult diet. **a** Larval dietary history regulates cuticular 7(Z)-tricosene levels in *white Dahomey* males at 3 weeks of age. LOW and STD flies were sorted into three weight bins, revealing that LOW heavy flies have significantly less 7(Z)-tricosene than STD light flies, despite being close in body size (*n* = 3 for LOW light and LOW medium, *n* = 5 for LOW heavy, *n* = 6 for STD light and STD medium, and *n* = 4 for STD heavy). **b** Adult diet influences cuticular 7(Z)-tricosene levels in STD *iso31* males at 3 weeks of age. The levels of the four major male cuticular hydrocarbons are higher on HYHG than on LY, HY or HG adult diets (*n* = 6). ***p < 0.001, two-way ANOVA followed by the Bonferroni multiple comparisons test. Error bars show 1 s.d.

The results thus far raise the question of whether developmental diet has a long-term influence upon the oenocyte synthesis of alkenes, alkanes or both during adult life. As with body size, adult oenocyte size was smaller in LOW than in STD flies but no differences in cell morphology were apparent (Fig. 7a). In addition, oenocyte size in both LOW and STD animals was smaller for flies maintained on the adult LYHG than on the HYHG diet (Fig. 7a). Hence, the yeast concentration of both the developmental and the adult diets can influence the size of adult oenocytes. We next investigated whether developmental diet can affect adult oenocyte function, as reflected by hydrocarbon synthesis. LOW flies produced similar amounts of the non-oenocyte pheromone cVA as STD flies but ~60% of the tricosane and only ~30% of the 7(Z)-tricosene (Fig. 7b, Supplementary Fig. 3a, b). This indicates that the hydrocarbons of LOW flies do not simply scale down with the 50% decrease in their body mass: rather there is a more substantial decrease in alkenes, thus lowering the C23 and C25 alkene:alkane ratios (Fig. 7c, d). Similarly, for the heterogeneous *w* ^*Dah*^ strain, the largest LOW flies had substantially less 7(Z)-tricosene per fly (Fig. 8a) and lived longer than the smallest STD flies, despite their roughly similar body sizes (Fig. 2c–g). Not only developmental diet but also adult diet influences cuticular hydrocarbon levels such that they are highest on HYHG, the diet with the greatest difference in survival between LOW and STD flies (Fig. 8b). In principle, LOW and STD animals could also differ in their resistance to autotoxins but, although LOW live longer than STD flies on HYHG, their survival curves overlap in vials supplemented with 125 μg synthetic 7(Z)-tricosene (Fig. 7e). Hence, 7(Z)-tricosene shortens the median lifespan of LOW more than STD flies (54% vs. 30%) but, in absolute terms, their survival is roughly comparable. It is nevertheless possible that STD flies have higher levels of endogenous alkenes on their cuticle than LOW flies and so could be exposed to a higher total concentration of autotoxins in the vial supplementation assay. In summary, these results demonstrate that larval yeast restriction influences adults to produce a less toxic blend of hydrocarbons. In turn, this would be predicted to enhance survival in adult environments where autotoxins are limiting for lifespan.

### Housing density influences lifespan via alkene autotoxins

**Fig. 9:**
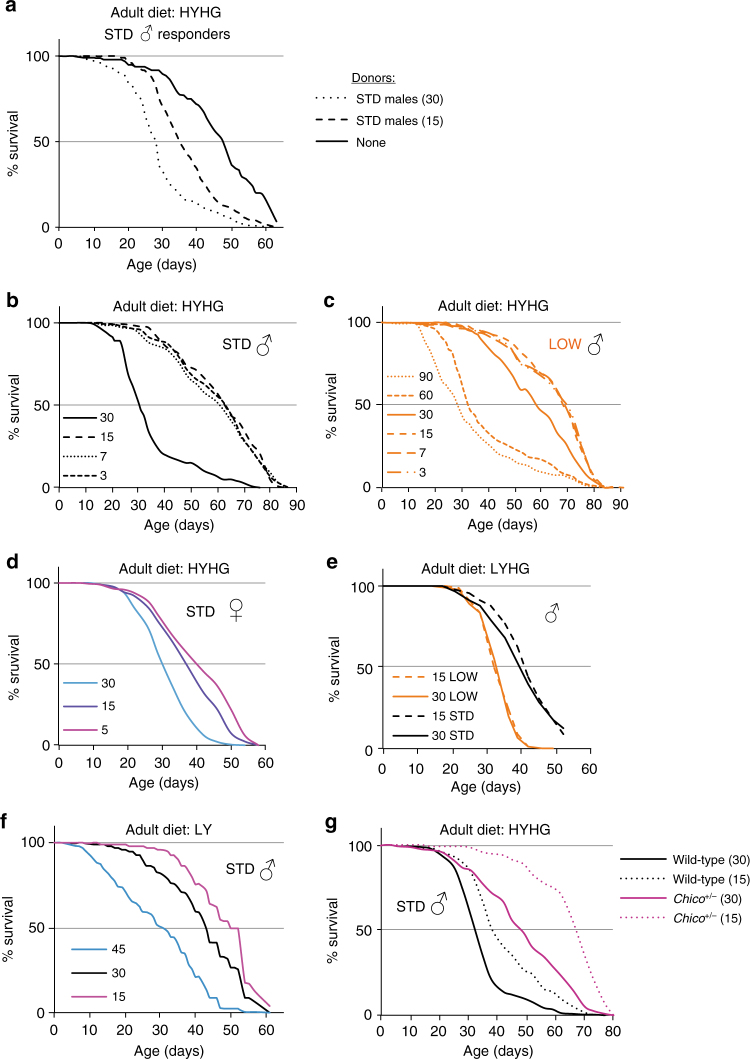
Effects of housing density, diet and *chico* heterozygosity upon lifespan. **a** Survival of STD responder males in vials preconditioned with 15 or 30 STD donor males (15 responders per vial). Note that the survival curves for no donors and 30 STD male donors are the same as those shown in Fig. 4d as these experiments were done in parallel. **b**–**d**, Survival of STD males **b** LOW males **c** and STD females **d** on HYHG diet at housing densities ranging from 3 to 90 flies per vial. **e** Survival of STD and LOW males on LYHG diet at 15 or 30 flies per vial. **f** Survival of STD males on LY diet at 15, 30 or 45 flies per vial. **g** Survival of *chico* ^+/−^ and *chico* ^+/+^ STD males, housed on HYHG at densities of 15 or 30 per vial

We next investigated whether autotoxins might underlie the mechanism-of-action of other factors known to influence *Drosophila* lifespan. An increase in population density can decrease lifespan and, although reported nearly a century ago^44–46^, the underlying mechanism remains unknown. Our vial pre-conditioning paradigm indicates that halving the number of donor flies per vial from 30 to 15 generates less autotoxin activity (Fig. 9a). We next measured male and female lifespans on the adult HYHG diet at densities ranging from 3 to 90 flies per vial, confirming that lower densities tend to correlate with markedly increased lifespans for both LOW and STD animals (Fig. 9b–d). At low densities, the Kaplan–Meier survival curve approaches a limit at which further density dilutions no longer extend lifespan. We use the term neutral density to describe the maximum number of flies per vial at which this lifespan limit is sustainable. Thus, on the HYHG adult diet, the neutral densities for STD males and STD females are between 15–30 and 5–15 respectively. Comparing STD and LOW males, the neutral density of both lies somewhere between 15 and 30 but median lifespan at 30 is shortened more for STD than for LOW flies (Fig. 9b, c). This points to the neutral density of LOW flies being higher than that of STD flies. The magnitude of the lifespan extension induced by lowering housing density is greatly influenced by the yeast and glucose content of the adult diet (Fig. 9b, e, f). On one adult diet, LYHG, decreasing housing density from 30 to 15 did not increase lifespan at all (Fig. 9e). This was also the only adult diet on which LOW fared worse than STD flies in terms of high adiposity and median lifespan (Fig. 1b, c). Together, the above results raise the possibility that housing density, like developmental dietary history, might influence lifespan via autotoxins. To test directly whether oenocyte alkenes are required for housing density effects upon lifespan, we analysed the survival of STD *desat1* knockdown and control flies housed at different densities. Halving the housing density from 30 to 15 markedly increased the lifespan of controls but, importantly, not of *desat1* knockdown flies (Fig. 7f). This striking result indicates that the negative effect of high population density upon lifespan requires oenocyte-specific *desat1* activity and so is likely to be mediated by autotoxic oenocyte alkenes. Mortality analysis of our survival curves provides hints that housing density, autotoxins and developmental dietary history may all impact ageing in a similar way, altering age-dependent demographic frailty (Supplementary Fig. 4, Supplementary Data 2). With regard to housing density, we also note that the difference between the median lifespans of wild-type LOW and STD flies is much smaller below neutral density than above it (Fig. 9b, c). This strongly suggests that autotoxins make a significant contribution towards the regulation of longevity by developmental dietary history.

### Decreased insulin signalling alters the autoxin response

Given that insulin signalling is a key regulator of longevity^22–24^, we investigated whether it interacts with alkene autotoxins. Heterozygosity for *chico*, encoding an insulin receptor substrate (IRS) orthologue, does not alter developmental timing or body size but it is known to extend *Drosophila* lifespan and to increase xenobiotic resistance, although these two effects can be experimentally uncoupled^47–49^. We found that *chico* heterozygous and wild-type males produce almost identical amounts of autotoxin activity and that they also have very similar cuticular hydrocarbon profiles (Fig. 7g, h). Nevertheless, doubling the housing density of *chico* males decreases median lifespan by a larger percentage than that of wild-type males (30% vs. 16%) although, in absolute terms, *chico* flies still survive longer (Fig. 9g). Similarly, the addition of 7(Z)-tricosene decreases the median lifespan of *chico* males by a slightly larger percentage than that of wild-type males (50% vs. 42%) but, in absolute terms, *chico* again survive longer than wild-type flies (Fig. 7i). These results together demonstrate that survival in the presence of autotoxins is sensitive to the level of insulin signalling.

## Discussion

This study shows that dietary yeast restriction during *Drosophila* development can induce long-term changes in adult triglyceride storage, xenobiotic resistance and lifespan. It can also extend lifespan even when adults are switched to a high yeast diet. In contrast, longevity obtained via adult-onset dietary restriction (DR) is largely reversible upon switching to a non-restricted diet^50^. Developmental-diet induced extensions of median lifespan can be as large or larger than those observed with adult DR but this depends strongly upon the adult environment. Hence, it is the combination of developmental and adult environments that determines survival outcomes, not one or the other. A major finding of this study is that male and female flies condition their environment with alkene autotoxins that decrease the survival of both adult sexes. Developmental yeast restriction influences adult oenocytes to synthesise a hydrocarbon blend that contains a lower proportion of alkene autotoxins. In turn, this promotes increased longevity in many adult environments but not those where lifespan is limited by other toxic factors, such as paraquat or a high glucose-to-yeast ratio diet. Alkenes appear to have a selective mechanism-of-action as physiological amounts of tricosene kill adult flies but not larvae. Their influence upon *Drosophila* adults is far-reaching and affects not only how survival is regulated by developmental dietary history but also by population density, sex and insulin signalling. This has important implications for laboratory lifespan studies, with our results suggesting that the autotoxin contribution can be teased apart from other mechanisms by measuring survival curves below neutral density, a parameter that will need to be measured case-by-case. In more natural environments, the contributions of autotoxins to ecology are not yet known but we note that *Drosophila* congregate at high density on rotting fruit^51^ and so may be exposed to harmful levels of alkenes. During evolution, the selective advantages conferred by alkenes as sex pheromones, barrier lipids and/or mediators of other beneficial activities are likely to have outweighed any disadvantages due to decreased longevity.

It is surprising that a major class of *Drosophila* autotoxins corresponds to lipids on the body surface, some of which are known to act as sex pheromones. This link is also emerging in *Caenorhabditis elegans*, where recent work shows that male pheromone contains ascaroside lipids that mediate the density-dependent mortality of grouped males and that shorten the survival of both sexes^52, 53^. Furthermore, although the molecules involved are different, plants also synthesise toxic metabolites (allelochemicals) and these can suppress nearby plants of the same or of a different species^54^. There are strong similarities between insect and mammalian integument (skin) lipids. They include unsaturated species that are dependent upon Δ9 stearoyl-CoA desaturases, such as Desat1 in *Drosophila* and SCD1 in mammals^38, 55^. Furthermore, integument lipids contain alkenes such as tricosene in *Drosophila* and birds, and squalene in humans^33, 56, 57^, both of which can decrease *Drosophila* survival in our supplementation assays. We also highlight a particularly striking analogy between specialised subepidermal cells in insects (adult oenocytes), birds (preen gland cells) and mammals (sebocytes), which each secrete complex mixes of integument lipids with beneficial barrier functions^33, 58^. Future studies will be needed to determine whether physiological amounts of skin-derived lipids can influence longevity in mammals, as they do in *Drosophila*.

## Methods

### *Drosophila* strains

*Drosophila* were raised at 25 °C unless otherwise stated. The control strain used, unless otherwise stated, was *iso31*, an isogenic strain selected for wild-type viability, fertility and behaviour, corresponding to *w* ^*1118*^_*iso*_; *2*_*iso*_; *3*_*iso*_ line 31^59^. *Wolbachia* was cleared from the original *iso31* line using standard food supplemented with 50 μg/mL tetracycline for 4 generations and its absence confirmed by PCR. *Wolbachia*-negative *iso31* was then reared on standard diet for at least 10 generations before experiments were initiated. *Wolbachia*-negative and positive *iso31* lines displayed similar developmental survival and lifespans on larval low yeast diet. *iso31* flies were negative for *Wolbachia* in all experiments except for Fig. 1 and Supplementary Fig. 1. Non-isogenic wild-type flies in this study were *Oregon*-*R* or *white Dahomey* (*w* ^*Dah*^; gift from Matthew Piper), a *w* ^*1118*^ outbred laboratory strain^60^. Chico heterozygotes and their corresponding controls (*y* ^*1*^;*cn* ^*1*^,*chico* ^*1*^/*cn* ^*1*^;*ry* ^*506*^ and *y* ^*1*^;*cn* ^*1*^;*ry* ^*506*^, respectively) were isogenic for genetic background^48^. Other genetic elements used were: *PromE* ^*TS*^ (*PromE(800)-GAL4*, *tub-GAL80* ^*ts*^, *UAS-mCD8∷GFP/(Cyo,Dfd::YFP)* ^61, 62^, *UAS-TSC1 + 2* (gift from Nic Tapon), *UAS-desat1* RNAi (VDRC line GD33338) and *UAS-Cyp4g1* RNAi^63^. Δ*ppk23* and Δ*ppk29* deletion mutants were a gift from Kristin Scott^41^. For genetic manipulations using the PromE^TS^ driver, control genotypes were generated by crossing the driver with *w* ^*1118*^ wild-type flies (Figs. 3d, 4d–f, 5f) or *yw* wild-type flies (Fig. 4b, c). For the survival curves for *PromE* ^*TS*^ > *desat1 RNAi* in Fig. 5f, the controls were not isogenic so comparisons should be made between adults of the same genotype at different housing densities, not between different genotypes. Additional fly strains used in this study were obtained from the Bloomington *Drosophila* Stock Center at Indiana University and the Vienna *Drosophila* Resource Center.

### Diets and dietary manipulations

All larval and adult diets contained cornmeal-glucose-yeast-agar and antimycotic agents and were prepared by boiling not autoclaving. All fly lines were reared on standard (STD) diet for at least 10 generations prior to experiments. STD diet (~2% w v^−1^ yeast) consists of 7.02 g agar (Brian Drewitt), 58.5 g glucose (VWR, Cat. No. 10117HV), 66.3 g cornmeal (Brian Drewitt), 23.4 g autolysed yeast extract (Brian Drewitt) and 19.5 mL of antimycotic solution containing 0.04% Bavistan (Sigma-Aldrich, Cat. No. 378674) and 10% Nipagin (Sigma-Aldrich, Cat. No. H3647) per litre. LOW is identical to STD diet but contained 1.17 g autolysed yeast extract per litre (~0.1% w/v yeast) for the *iso31* strain. For *w* ^*Dah*^, *chico* ^+/−^ and *chico* ^+/+^ strains LOW corresponds to 2.34 g autolysed yeast extract per litre (~0.2% w/v yeast), as larvae of these genetic backgrounds failed to develop on the 0.1% yeast version. The concentration of yeast in the LOW diet needed to obtain the ~50% decrease in adult body mass used in this study may need to be determined empirically as we sometimes observe batch-to-batch differences, perhaps due to variations in the cornmeal or other supplied ingredients. Larvae reared on LOW diets are developmentally delayed by at least 4 days at 25 °C. All adult diets contained 6.6% (w/v) cornmeal but glucose and autolysed yeast were varied: yeast was either 0.59% (LY), 2.34% (standard) or 9.36% (HY) and glucose was either 40% (HG) or 5.85% (standard). Larvae were reared at a controlled low density by seeding 250 mL plastic bottles (containing ~50 mL diet) with a fixed volume (13 μL) of phosphate buffered saline (PBS; Gibco)-washed embryos, obtained from timed collections of ~5–10-day-old parents fed on grapejuice-agar plates supplemented with live yeast paste. Due to differences in the rate of development, embryos used to generate LOW adults were collected 3–5 days earlier than those used for STD controls so that both groups eclosed within the same 48-h window.

### Measurement of body mass and adiposity

For body mass measurements, flies of ≥ 48 h after eclosion were collected under mild CO_2_ anaesthesia, transferred individually or in groups into pre-tared 2 mL tubes and weighed on a microbalance (Sartorius MSE3.6 P). For separation of *w* ^*Dah*^ flies into weight bins, males were first separated under mild CO_2_ anaesthesia from females, and after ~24 h, they were again mildly anaesthetised and individually weighed on a microbalance, transferring them by gently grasping onto the wings with blunt forceps. Body water content was determined as the difference between wet body mass and dry mass after heating to 65 °C for 72 h. For adiposity measurements, 15 STD or 30 LOW adult males at 2 weeks of age were frozen on dry ice after initial weighing and then stored at −80 °C. Lipids were extracted essentially as described^64^ with 500 μL of 0.9% NaCl solution in ddH_2_O and a ball-bearing (Retsch, ϕ6 mm) added to each sample prior to homogenisation in a ball-mill homogeniser (Retsch, MM301) for 2 min at 30 Hz. The homogenate was then transferred to a 10 mL glass extraction tube and the 2 mL tube rinsed with a further 250 μL 0.9% NaCl. Lipids were extracted in chloroform:methanol containing butyrated hydroxytoluene (BHT; 100 μg/mL) and internal standards (tripentadecanoin for trigylcerides and cholesteryl heptadecanoate for cholesteryl esters). The organic phase was transferred to a fresh glass tube and evaporated to dryness at 50 °C under nitrogen. Neutral lipids (cholesteryl esters and triglycerides) were purified by solid phase extraction on aminopropyl silica columns (Biotage; Insolute 100 mg NH2 sorbent 470-0010-A) following established protocols^65, 66^. Fatty acid methyl esters (FAMEs) were prepared by incubation in methanol containing 1.5% H_2_SO_4_ and analysed on an Agilent 7890A-5975C GC-MS system. Split injection (10:1, split flow 30 mL/min, injection temperature 250 °C) onto a 30 m × 0.53 mm × 1 μm Innowax column (Thames Restek, Saunderton, UK) was used with helium as the carrier gas. The initial oven temperature was 50 °C (1 min), followed by temperature gradients to 190 °C at 20 °C/min held for 6 min, from 190 to 210 °C at 4 °C/min held for 5 min, and from 210 to 240 °C at 10 °C/min. The final temperature was held for 10 min. Data analysis was performed using ChemStation software (version D.01.02.16, Agilent Technologies). FAMEs were identified and quantified based on comparison to retention times and spectra of authentic FAME standards (Sigma-Aldrich), followed by normalisation to the measured abundance of internal standards (tripentadecanoin for triglycerides and cholesteroyl heptadecanoate for cholestryl esters) to account for losses during sample preparation.

### Lifespan and vial preconditioning assays

Lifespan assays were performed largely as described^67^ and larvae and adults were reared at 25 °C unless otherwise stated. Adults eclosing over a 24 h period were transferred, without anaesthesia, into 250 mL bottles containing ~50 mL STD diet. After 48 h, once-mated adults of the appropriate sex were collected under mild CO_2_ anaesthesia and transferred to the specified adult diet in large polystyrene vials (28.5 mm O.D. × 95 mm H) stoppered with Flugs (Dutscher Scientific, Cat. No. 789035). Adults were reared in incubators at 25 °C with 60 ± 5% relative humidity and, twice per week, transferred to fresh food vials and deaths recorded. UAS transgene expression was restricted to adult stages using *tub-GAL80* ^*TS*^ ^62^: animals were raised at 18 °C until adulthood, switched at 0–24 h after eclosion to 29 °C for 48 h, and then once-mated adults were collected and switched to 25 °C for the remainder of the experiment. For vial preconditioning, donor flies were transferred and deaths recorded as for lifespan analysis. Dead donors were removed from preconditioned vials using clean forceps and responder flies were then added on the same day and typically within 2 h. Autotoxin activity in preconditioned vials was not detectably diminished after donor removal for 24 h. Donors were 3–10 days old and the first data point on survival curves indicates the age of responders at the start of the preconditioning assay. Unless otherwise stated, preconditioning assays used 30 donor and 15 responder flies and the age of responders at the start of the experiment is indicated by the age at which the survival curve begins.

### Vial and cuticle washes and supplementation

The food surface of vials was extracted with 250 μL of miliQ water or n-hexane for 30 s with gentle agitation and minimal tilting, so as to avoid washing the inner polystyrene surface of the vial. The rinse was then removed with a pipette tip (organic solvent-resistant for hexane experiments) and the washed vials allowed to dry unstoppered, typically for 0.5–2 h for hexane-extracted and overnight for water-extracted vials. For vial supplementation with cuticular hexane extracts, STD males housed on HYHG (30 per vial) for 2 weeks (Fig. 5b) or 3 weeks (Fig. 6f) were extracted in batches (180 flies in 9 mL hexane) for 1.5–2 min with gentle agitation. Due to early adult lethality*, PromE* > *Cyp4g1i* flies were only aged for 1 week (30 per STD vial) as were their corresponding *PromE* > *w* controls (Fig. 6d). Cuticular hexane extracts were transferred to clean glass vials, the hexane evaporated under nitrogen, and stored at −20 °C. The contents of each glass vial were resuspended in 2 mL n-hexane and 250 μL per vial was used, ensuring even coverage of the food surface. Similarly, for vial supplementation with synthetic lipids, fresh stocks in n-hexane were prepared at concentrations such that 250 μL of the solution was pipetted per vial. Synthetic lipids used in this study were: 7(Z)-tricosene (Cayman Chemical Cat. No. 9000313), 9(Z)-tricosene (Sigma-Aldrich Cat. No. 859885), tricosane (Sigma-Aldrich Cat. No. 263850), 7(Z)-pentacosene (Cayman Cat. No. 9000530), 7(Z),11(Z)-heptacosadiene (Cayman Chemical Cat. No. 10012567), heptacosane (Sigma-Aldrich Cat. No. 51560), 7(Z),11(Z)-nonacosadiene (Cayman Chemical Cat. No. 9000314), squalene (Sigma-Aldrich Cat. No. S3626), squalane (Sigma-Aldrich Cat. No. 85629), heptadecane (Sigma-Aldrich Cat. No. 128503) and nervonic acid (Sigma-Aldrich Cat. No. N1514). For all supplementation experiments, hexane was evaporated from vials for 3–6 h in a fume cupboard before use in survival assays. Unless otherwise stated, supplementation assays used 15 responder flies and their age at the start of the experiment is indicated by the age at which the survival curve begins.

### Egg laying and larval survival assays

To measure egg laying, once mated *iso31* females were maintained at 25 °C on STD diet at 15 flies per vial for one week and then transferred without anaesthesia to vials of HYHG diet or STD diet, supplemented with either hexane or 250 μg of 7(Z)-tricosene as described above. Egg laying was recorded over 20 h each day for a total of 7 days. The mean number of eggs laid per female per hr was calculated from six biological replicates. To measure larval survival and time to pupariation, synchronised first instar larvae were transferred at 10 per vial to STD diet supplemented with either hexane or 7(Z)-tricosene as described above. Pupae were counted from six biological replicates per condition every 6 to 12 h until all animals had pupariated.

### Stress resistance assays

For starvation resistance, 2 week-old *iso31* males were transferred without anaesthesia into vials containing 10 mL of 0.8% agar/PBS and deaths scored after 15 h and then every 3-4 h. To test for resistance to paraquat, males aged on HYHG (15 per vial) for 1 week were transferred, without anaesthesia, to HYHG containing 20 mM N,N′-dimethyl-4,4′-bipyridinium dichloride (Sigma-Aldrich, Cat. No. 856177). Deaths were scored every day and surviving flies were transferred into fresh paraquat supplemented vials every 3 days. For ethanol resistance assays, 3-week-old males were transferred, without anaesthesia, to vials containing filter paper discs (Whatman, Cat. No. 1001 042) soaked with 5% sucrose ± 6% ethanol, and incubated for 14 h. Flies were then scored for negative geotaxis in fresh vials i.e., flies climbing above 5 cm within 20 s after being tapped down to the bottom were counted as “active”. For each group, eight vials containing 10–15 males were scored three times.

### NMR analysis of preconditioned vial and cuticular extracts

The food surface of blank vials or vials preconditioned by adult males for 72 h at 25 °C was extracted for 30 s with 500 μL hexane using gentle agitation and minimal tilting. Cuticular extracts were prepared from 100 adult males of the indicated genotype, batch extracted in 8 mL hexane for 1.5–2 min with gentle agitation. Vial or cuticular extracts were then transferred to clean glass vials, evaporated to dryness in a TurboVap (Zymark Biotage) under a nitrogen stream and stored at −20 °C. Prior to analysis, samples were resuspended in 180 μL deuterated chloroform (Sigma-Aldrich, Cat. No. 225789) containing internal standard (0.03% v/v tetramethylsilane; TMS) and transferred to NMR tubes (3 mm OD, Bruker Biospin AG, P/N Z112272). NMR spectra were recorded at 800 MHz ^1^H frequency using the *zg* pulse sequence with a 30 s interscan relaxation delay. Peak identities were assigned and concentrations evaluated by reference to spectra obtained for authentic standards of cVA, hydrocarbons, fatty acids and other lipids, supported by analysis of 2D ^1^H,^1^H- and ^13^C–^1^H correlated spectral data (standard Bruker DQF-COSY, TOCSY, HSQC pulse sequences) for selected samples. Peak integration in 1D spectra was performed in Topspin 3.2. Estimates of hydrocarbon quantities deposited per vial over 72 h were calculated by comparing NMR spectra of hexane extracts from preconditioned vials, non-preconditioned vials, and vials supplemented with 125 µg synthetic 7(Z)-tricosene either immediately prior to extraction or after 72 h incubation next to vials being preconditioned. All spectra were first normalised to the internal standard (TMS) and the NMR signal compared to that of standard samples containing known amounts of synthetic 7(Z)-tricosene.

### GC–MS analysis of cuticular hydrocarbons

For each sample, five flies at 3 weeks of age (1 week of age for *PromE* ^*TS*^ > *Cyp4g1i* and corresponding *PromE* ^*TS*^ > *yw* controls) were extracted with gentle agitation for 1 min in 50 μL hexane spiked with 0.1 mM octadecane as an internal standard in 2 mL glass GC vials. The cuticular hexane extract was then transferred to glass GC vials with inserts (Agilent Technologies, Cat No. 5182-0715 and 5183-2085, respectively) and immediately analysed by GC–MS on an Agilent 7890B-5977A GC–MS system in EI mode. Splitless injection (injection temperature 270 °C) onto a 30 m + 10 m × 0.25 mm DB-5MS + DG column (J&W, Agilent Technologies) was used, using helium as the carrier gas. The initial oven temperature was 50 °C (1 min), followed by temperature gradients to 150 °C at 10 °C/min, from 150 to 249 °C at 3 °C/min, from 249 to 300 °C at 10 °C/min with a hold time of 10 min, and from 300 to 325 °C at 10 °C/min and a hold time of 12 min. Sample running order was randomised using the MassHunter sample sequence randomiser. Data analysis was performed using MassHunter Quantitative Analysis software (Version B.06.00, Agilent Technologies). Where possible, hydrocarbons were identified and quantified based on comparison to retention times, spectra, and measured abundances of authentic standards (Sigma-Aldrich). Where authentic standards were not available, peaks were annotated based on their spectra or assigned as “unknowns”. In these cases, only relative quantification (compared to internal standard) was possible.

### Immunostaining and confocal microscopy

For adult oenocyte analysis, abdominal cuticle/epidermis preps were dissected in PBS, fixed in 4% methanol-free paraformaldehyde (TAAB) in PBS for 30 min, washed in PBS and permeabilised in 0.3% Triton X-100 in PBS (PBT) for 1 h. Tissues were then incubated with DAPI (1 mg/mL; Sigma-Aldrich, Cat. No. D9542) in PBT for 2 h at room temperature or overnight at 4 °C. Following washing in PBT, detergent was removed by multiple PBS washes overnight at 4 °C. Tissues were mounted in Vectashield (Vector Labs) and imaged using a Leica SP5 confocal microscope. Image processing was performed in Fiji and Adobe Photoshop CS5, and all images shown are maximum intensity projections. For Fig. 5d, dorsal oenocytes were imaged from once-mated males housed at 30 per HYHG vial until 1 week of age. For Fig. 7a, ventral oenocytes (V3 cluster) were imaged from once-mated males housed at 30 per vial on either HYHG or LYHG until 2 weeks of age.

### Statistical analysis

Survivorship was plotted using Microsoft Excel and analysed using OASIS^68^. Age-specific mortality rates were calculated as described previously^25^. Conclusions are based on at least two independent survival tests for replicated or closely related experiments. Sample sizes were determined based on previous experience and the literature. No statistical methods were used to predetermine sample sizes. Log-rank analysis was used to test for statistical significance. For data shown in Fig. 2b, Fisher’s exact test was also used. All other data were plotted and analysed in Prism 6 (GraphPad). Two-tailed *t*-tests were used to analyse data in Figs. 1b, 7b–d. Ethanol stress resistance data were analysed using ordinary one-way ANOVA followed by Tukey’s multiple comparisons test. Cuticular hydrocarbon data in Figs. 6b, c, e, 7h, 8, Supplementary Fig. 3b were analysed using two-way ANOVA followed by the Bonferroni multiple comparisons test. In all figures, error bars represent 1 s.d. and statistically significant differences are indicated on graphs as: *p < 0.05, **p < 0.01, ***p < 0.001. For survivorship analyses, sample sizes, median and maximum lifespans, and statistical significance are shown in Supplementary Data 1. For all experiments, individual flies were sorted randomly into different treatment groups. For biochemical analyses, the investigator received samples labelled with numerical ID only. Blinding was not possible for survival and some other analyses due to obvious differences in the body size of STD and LOW flies.

### Data Availability

The data sets generated for this manuscript are available from the corresponding author upon reasonable request and all survival data are provided in Supplementary Data 1, 2.

## Electronic supplementary material

Supplementary Information

Description of Additional Supplementary Files

Supplementary Data 1

Supplementary Data 2
