# Supplementary material for: Developmental diet regulates Drosophila lifespan via lipid autotoxins
Source: Nat Commun. 2017 Nov 9;8:1384. doi: 10.1038/s41467-017-01740-9 (PMC5680271; doi:10.1038/s41467-017-01740-9)
Supplement: Supplementary file 1 — Supplementary Information [file 41467_2017_1740_MOESM1_ESM.pdf]

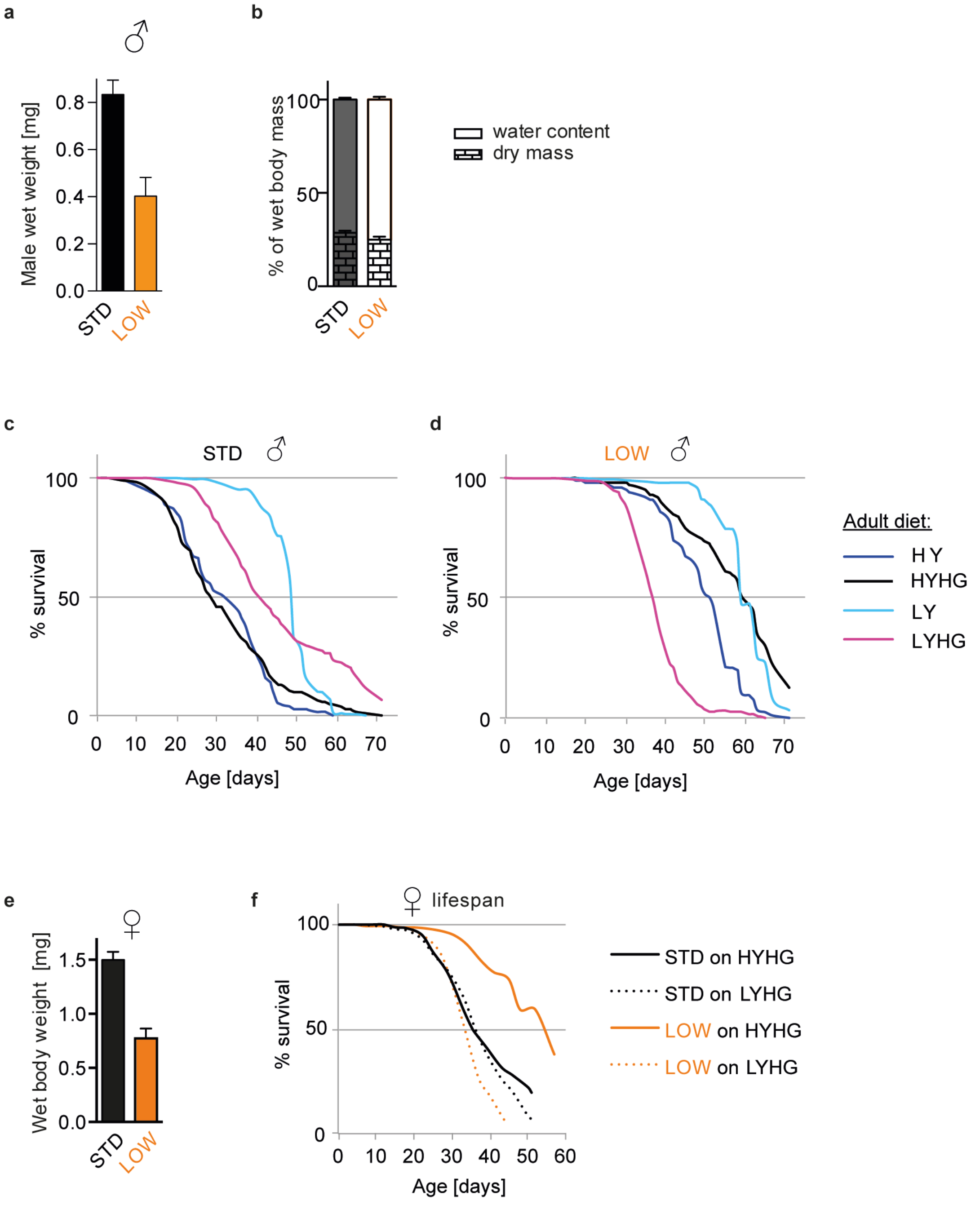

Supplementary Figure 1: Lifespan and body mass effects of larval dietary manipulations.

a, Wet body masses of individually-weighed STD (n=23) and LOW (n=23) *iso31* adult males aged for 1 wk on HYHG diet. **b**, Percentage dry body mass (hatched) and water content (unhatched) of STD and LOW *iso31* adult males aged for 1 wk on HYHG. In both cases: 20 males per sample, n=5 samples. **c**,**d**, Survival of STD **(c)** or LOW **(d)** adult males on four different adult diets. These survival curves are also shown in Fig. 1c, but are replotted here to allow comparisons between adult diets. **e**, Wet body masses of individually-weighed STD (n=7) and LOW (n=26) adult females aged for 1 wk on HYHG. **f**, Survival of STD or LOW adult females on HYHG or LYHG diets. For panels a, b and e: error bars show 1 s.d.

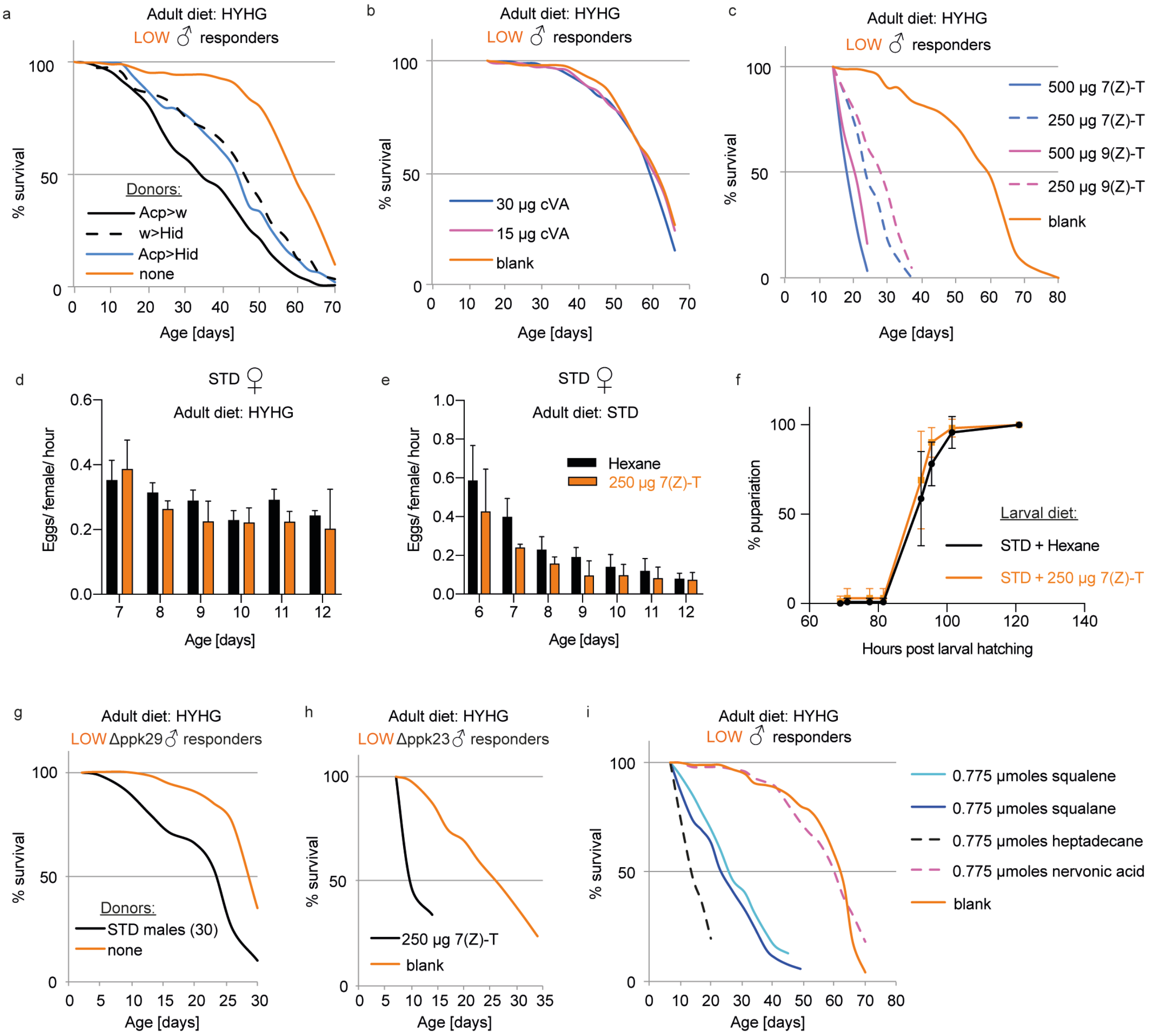

Supplementary Figure 2: The response to autotoxins does not require *ppk23* or *ppk29*.

a, Survival of LOW male responders is shortened to a similar extent by cohousing with STD donors of a control genotype (*w>hid* and *Acp>w*) or those expressing the pro-apoptotic gene *hid* in main cells of the accessory glands (*Acp>hid*) **b,** Survival of LOW males is not significantly altered by exposure to 15 or 30 µg of synthetic *cis*-vaccenyl acetate (cVA) compared to solvent alone (blank). **c**, Survival of LOW males is decreased by 250 or 500 µg of synthetic 7(Z)-tricosene or 9(Z)-tricosene, compared to hexane solvent alone (blank). **d-e,** Rate of egg laying by STD females at 1 wk of age on HYHG (d) or STD (e) adult diets is not affected significantly ($p>0.05$; two-way ANOVA followed by Sidak's multiple comparisons test) by exposure to 250 µg of synthetic 7(Z)-tricosene compared to hexane solvent alone ($n=3$ independent experiments and each experiment used 6 vials containing 15 females each). **f,** Larval time to pupariation and survival are not affected by supplementing STD larval diet with 250 µg of synthetic 7(Z)-tricosene (STD + 250 µg 7(Z)-T), compared to solvent alone (STD + hexane). $n=10$ vials containing 10 larvae each and one representative experiment of three independent experiments is shown. **g**, Survival of Δ*ppk29* males is decreased by autotoxins in vials preconditioned with 30 STD males. **h,** Survival of Δ*ppk23* males is decreased in vials supplemented with 250 µg synthetic 7(Z)-tricosene (7(Z)-T) compared to hexane solvent alone (blank). **i,** Survival is decreased by 0.775 µmol (molar equivalent of 250 µg 7(Z)-T) of squalene

(C30:6), squalane (C30:0) or heptadecane (C17:0) compared to nervonic acid (C24:1) or hexane solvent alone (blank). All survival curves used 15 LOW male responders per HYHG vial. Error bars show 1 s.d in panels d,e and f.

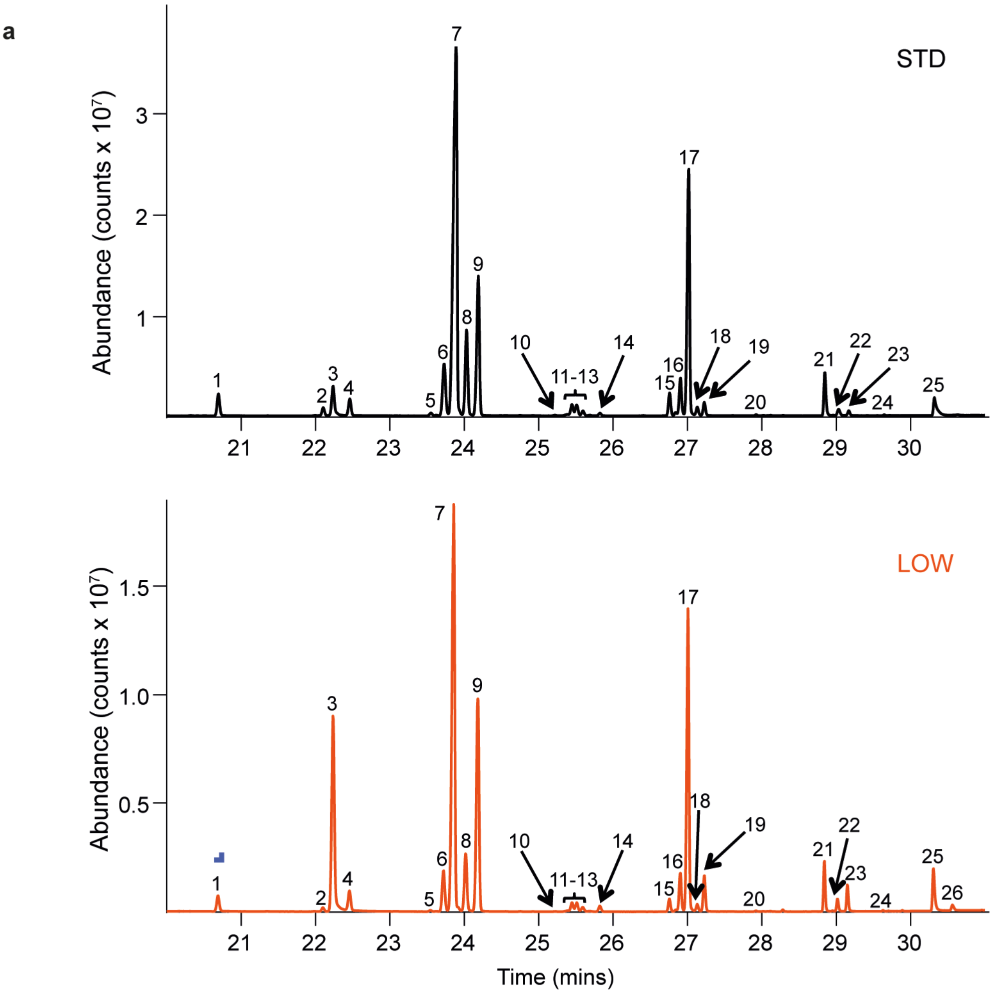

b

Peak no.	Hydrocarbon	STD	LOW
1	Heneicosane	0.34 ± 0.04	0.11 ± 0.03
3	11-cis-Vaccenyl Acetate	1.52 ± 1.28	1.97 ± 0.97
4	Docosane	0.21 ± 0.02	0.10 ± 0.02
6	9(Z)-Tricosene	1.45 ± 0.13	0.43 ± 0.12
7	7(Z)-Tricosene	14.58 ± 1.20	4.59 ± 1.02
9	Tricosane	1.75 ± 0.12	0.99 ± 0.19
14	Tetracosane	0.03 ± 0.00	0.02 ± 0.00
17	7(Z)-Pentacosene	5.32 ± 0.19	2.11 ± 0.54
19	Pentacosane	0.11 ± 0.01	0.10 ± 0.01
23	Heptacosane	0.03 ± 0.00	0.04 ± 0.01
26	Nonacosane	n.d.	0.01 ± 0.00

Supplementary Figure 3. GC-MS analysis of STD and LOW cuticular extracts.

a, Representative chromatograms from GC-MS analyses of cuticular hexane extracts from STD and LOW adult males on HYHG at 3 wks of age. Note the difference in y-axis scales between the two chromatograms. Key to peaks: 1, Heneicosane; 2, B-Docosene; 3, 11-cis-Vaccenyl acetate; 4, Docosane; 5, Unknown (281); 6, 9(Z)-Tricosene; 7, 7(Z)-Tricosene; 8, C-Tricosene; 9, Tricosane; 10, Unknown (295); 11, A-Tetracosene; 12, B-Tetracosene; 13, C-Tetracosene; 14, Tetracosane; 15, Unknown (309); 16, A-Pentacosene; 17, 7(Z)-

Pentacosene; 18, C-Pentacosene; 19, Pentacosane; 20, Unknown (323); 21, Unknown (337); 22, B-Heptacosene; 23, Heptacosane; 24, Unknown (351); 25, Unknown (365); 26, Nonacosane. Definitive identifications were assigned to peaks only if this was verified by direct comparison to a pure synthetic standard. A letter at the start of the name of an unsaturated hydrocarbon denotes that the position of the double bond is not known. 'Unknowns' are unidentified hydrocarbon-like molecules, with their diagnostic ion in brackets. Note that all peaks except cVA (peak 3) are absent or severely reduced in adult males expressing *Cyp4g1* RNAi under the control of *PromE^{TS}-GAL4*[37]. **b**, Quantifications of identified cuticular hydrocarbons are expressed as abundance normalised to an internal standard (a.u.) ± 1 s.d. n=6 for STD and LOW flies. In all cases, extracts were prepared from 5 male flies per sample.

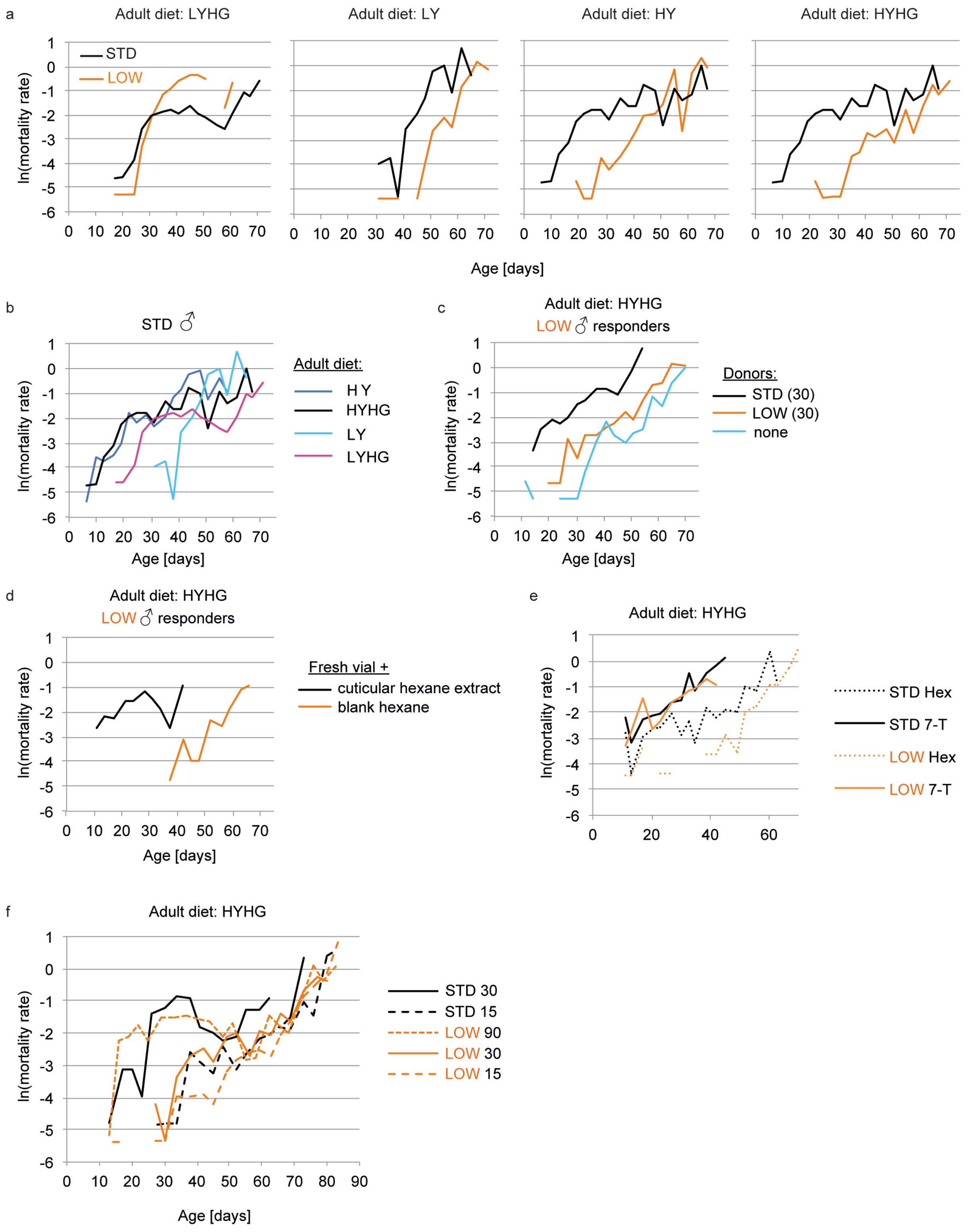

Supplementary Figure 4. Age-specific mortality analysis for the effects of diet, autotoxins and housing density. Ln (mortality rate) plots for survival data shown in Fig. 1c (panel **a**), Supplementary Fig. 1c (panel **b**), Fig. 3g (panel **c**), Fig. 5b (panel **d**), Fig. 7e (panel **e**) and Fig. 9b, c (panel **f**).
