# Supplementary material for: Developmental diet regulates Drosophila lifespan via lipid autotoxins
Source: Nat Commun. 2017 Nov 9;8:1384. doi: 10.1038/s41467-017-01740-9 (PMC5680271; doi:10.1038/s41467-017-01740-9)
Supplement: Supplementary file 2 — Description of Additional Supplementary Files [file 41467_2017_1740_MOESM2_ESM.docx]

**Description of Additional Supplementary Files**

File Name: Supplementary Data 1

Description: Cohort sizes, median and maximum lifespans, and log-rank tests for Kaplan-Meier survival curves in this study.

File Name: Supplementary Data 2

Description: Life tables used for Supplementary Figure S4.
